# Seasonal Changes in *Thrips tabaci* Population Structure in Two Cultivated Hosts

**DOI:** 10.1371/journal.pone.0101791

**Published:** 2014-07-03

**Authors:** Brian A. Nault, Wendy C. Kain, Ping Wang

**Affiliations:** Department of Entomology, Cornell University, New York State Agricultural Experiment Station, Geneva, New York, United States of America; Rutgers University, United States of America

## Abstract

*Thrips tabaci* is a major pest of high-value vegetable crops and understanding its population genetics will advance our knowledge about its ecology and management. Mitochondrial cytochrome oxidase subunit I (COI) gene sequence was used as a molecular marker to analyze *T. tabaci* populations from onion and cabbage fields in New York. Eight COI haplotypes were identified in 565 *T. tabaci* individuals collected from these fields. All *T. tabaci* were thelytokous and genetically similar to those originating from hosts representing seven plant families spanning five continents. The most dominant haplotype was NY-HT1, accounting for 92 and 88% of the total individuals collected from onion fields in mid-summer in 2005 and 2007, respectively, and 100 and 96% of the total in early fall in 2005 and 2007, respectively. In contrast, *T. tabaci* collected from cabbage fields showed a dynamic change in population structure from mid-summer to early fall. In mid-summer, haplotype NY-HT2 was highly abundant, accounting for 58 and 52% of the total in 2005 and 2007, respectively, but in early fall it decreased drastically to 15 and 7% of the total in 2005 and 2007, respectively. Haplotype NY-HT1 accounted for 12 and 46% of the total in cabbage fields in mid-summer of 2005 and 2007, respectively, but became the dominant haplotype in early fall accounting for 81 and 66% of the total in 2005 and 2007, respectively. Despite the relative proximity of onion and cabbage fields in the western New York landscape, *T. tabaci* populations differed seasonally within each cropping system. Differences may have been attributed to better establishment of certain genotypes on specific hosts or differing colonization patterns within these cropping systems. Future studies investigating temporal changes in *T. tabaci* populations on their major hosts in these ecosystems are needed to better understand host-plant utilization and implications for population management.

## Introduction

*Thrips tabaci* Lindeman is a major insect pest of multiple crops worldwide including two high-value vegetable crops, onion, *Allium cepa* L., and cabbage, *Brassica oleracea capitata* (L.) [1]. *Thrips tabaci* is known to have multiple biotypes with different reproduction modes, host preferences and virus-transmission competencies [2]–[6]. Genetic analysis using molecular markers suggests that *T. tabaci* is a complex of cryptic species [7]. Genetic differences between *T. tabaci* populations from tobacco and populations from onion and leek have been documented [7], [8]. Understanding the genetic structure of *T. tabaci* populations in cropping systems will advance our knowledge about its population ecology, which can be important for developing effective management strategies [9].

In New York (USA), *T. tabaci* infestations in onion and cabbage fields are routinely managed with applications of insecticides. Insecticide resistance in *T. tabaci* populations in onion fields has been widespread [10], [11]. In contrast, control failures with similar insecticides have not been reported in New York cabbage fields, suggesting that populations in cabbage fields remain susceptible to these products. For example, neonicotinoid insecticides such as imidacloprid and acetamiprid continue to effectively control populations of *T. tabaci* in cabbage [12], but have been ineffective against *T. tabaci* infestations in onion [13]–[15]. In addition, seasonal susceptibility shifts in *T. tabaci* populations in onion fields to insecticides have also been documented [10], [11]. Some populations were susceptible to insecticides in mid-summer, but tested resistant to them in early fall, and vice versa. These observations suggest that there may be host-associated differences among *T. tabaci* populations and that they may change during the season.

Knowledge about *T. tabaci* population genetics is important for developing management strategies, especially those that mitigate insecticide resistance. Mitochondrial DNA markers have been successfully used to identify genetic differentiation among *T. tabaci* populations [4]–[7], [16], [17]. In our study, molecular markers from the mitochondrial cytochrome oxidase subunit I (COI) gene were used to analyze *T. tabaci* populations collected from onion and cabbage fields in New York to examine 1) the differentiation between *T. tabaci* populations from these crops and how they relate to conspecifics from other reproductive modes, hosts and locations, and 2) seasonal changes in *T. tabaci* population genetic structure from onion and cabbage cropping systems. Our results revealed a commonality in the most abundant *T. tabaci* haplotypes from onion and cabbage fields and these haplotypes were genetically similar to those originating from an array of hosts and locations around the world. All haplotypes from onion and cabbage fields were in the same lineage as those that reproduce via thelytoky; none were in the arrhenotokous or “tobacco type” clades. The population genetic structure of *T. tabaci* populations in onion fields was similar between mid-summer and early fall, whereas the population structure in cabbage fields changed substantially between these periods.

## Materials and Methods

### Ethics statement

Vegetable growers gave us verbal permission to collect thrips on their privately owned land and no permits were required to collect this common insect. Sampling did not involve regulated, endangered or protected species.

### Insect collection

**Figure 1:**
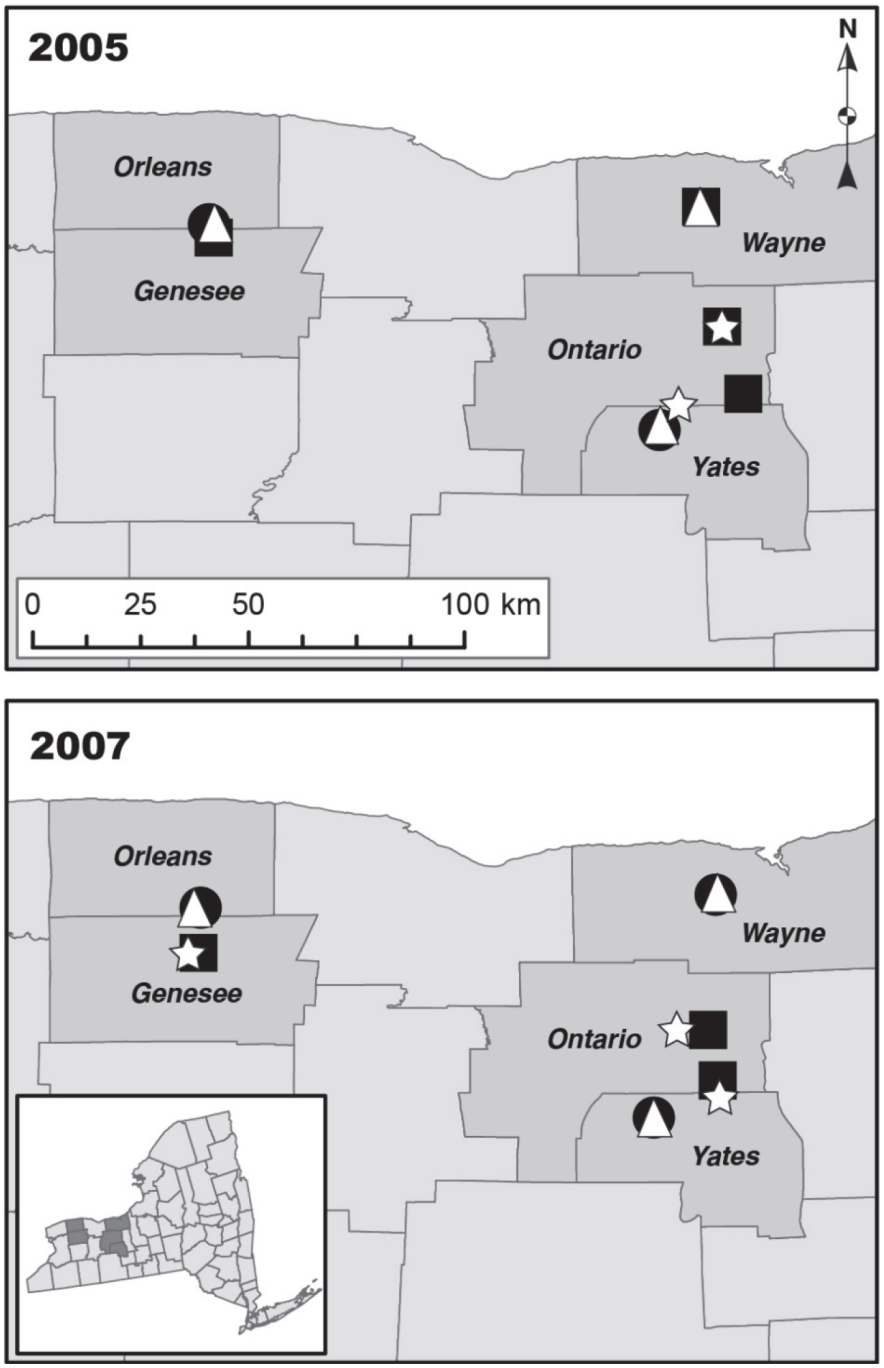
Map of onion and cabbage fields where *Thrips tabaci* adults were sampled. The following symbols indicate the season and crop sampled: • = mid-season, onion; Δ early fall, onion; ▪ = mid-season, cabbage; * = early fall, cabbage.

*Thrips tabaci* adults were collected from commercial onion and cabbage fields in western New York in 2005 and 2007. Onion and cabbage fields were sampled from two of the largest onion and cabbage production regions in New York (Figure 1). Regions were separated from one another by at least 48 km and onion and cabbage fields within a region were separated by a minimum of 3.2 km. The landscape surrounding onion and cabbage fields differed. Onion fields were located on muck soil and ranged in size from 2 to 5 ha. These fields were bordered by several to many other onion fields in contiguous plantings that ranged from approximately 40 ha in Wayne Co., 240 ha in Yates Co, and 1,000 ha in Orleans/Genesee Counties. These onion monocultures were all bordered by woods. Cabbage fields were located on mineral soil and were often bordered by other crops such as corn, soybean, alfalfa and small grains and occasionally woods. Cabbage fields ranged from 12 to 20 ha.

*Thrips tabaci* populations were sampled from onion and cabbage fields during mid-summer and early fall. In mid-summer 2005 and 2007, sampling occurred between July 15–29 and July 17–26, respectively. In early fall 2005 and 2007, sampling occurred between Sept 8–19 and Sept 5–19, respectively. In 2005 and 2007, two of the same onion fields were sampled in mid-summer and early fall. The third onion field in each year was harvested early, so an adjacent onion field was sampled in early fall (Figure 1). One cabbage field was sampled both in mid-summer and early fall in 2005, whereas all others in 2005 and 2007 were harvested in August and cabbage fields in relative proximity to each other (e.g., within 3.2 km) were selected for sampling in early fall (Figure 1).

No more than one adult thrips was sampled per plant and plants were at least 1.5 m apart. Adults were removed using a fine-tipped paintbrush and then placed into vials containing 95% ethanol; adults from the same field and sampling period were placed into the same vial. The total number of *T. tabaci* adults sampled was 229 and 336 in 2005 and 2007, respectively. In onion, 129 and 165 adults were sampled in 2005 and 2007, respectively; in cabbage, 100 and 171 adults were sampled in 2005 and 2007, respectively. Voucher specimens are located at Cornell University’s Department of Entomology, New York State Agricultural Experiment Station, Geneva, NY.

### PCR amplification of a *COI* fragment from *Thrips tabaci*

Thrips preserved in ethanol were rinsed with de-ionized water to remove ethanol and then DNA from individual thrips was isolated with a rapid genomic DNA preparation method [18]. A single thrips was ground in 10 µl of lysis buffer containing 50 mM KCl, 2.5 mM MgCl_2_, 0.45% Nonidet P-40, 0.45% Tween 20, 0.01% gelatin and 60 µg/ml protease K in 10 mM Tris-HCl (pH 8.3), and incubated at 65°C for 30 min and then heated at 95°C for 15 min to inactivate the protease K activity in the lysate. The lysate was used as the DNA template for PCR amplification of a 706 bp fragment of mtCOI, using the universal COI primer pair for insects, LepF: ATTCAACCAATCATAAAGATATTGG and LepR: TAAACTTCTGGATGTCCAAAAAATCA [19]. Twenty five µl PCR reactions were prepared to contain 1 µl of thrips DNA prepared above, 2.5 U of Taq DNA polymerase (New England Biolabs, Beverly, MA), 0.4 mM of dNTPs, 0.4 µM of each primer and 2.5 µl of 10x Taq DNA polymerase buffer (New England Biolabs, Beverly, MA). The PCR reaction was performed by a denaturation incubation at 94°C for 1 min, followed by 40 cycles of 94°C for 30 sec, 52°C for 30 sec, and 72°C for 40 sec, and a final extension at 72°C for 10 min. The PCR products from each individual thrips were examined by 1% agarose gel electrophoresis to confirm the correct amplification of the COI fragment from the individuals analyzed.

### DNA sequencing and sequence analysis

The PCR products were purified for DNA sequencing using a one-step enzymatic purification method [20]. Five µl of PCR product was mixed with 1 µl of enzyme solution containing 0.5 unit of shrimp alkaline phosphatase (USB, Cleveland, OH) and 1.25 unit of exonuclease I (USB) and incubated at 37°C for 60 min, followed by incubation at 90°C for 10 min to inactivate the enzymes. The enzyme-treated PCR products were sequenced using the BigDye Terminator v3.1 Cycle Sequencing Kit (Applied Biosystems, Foster City, CA) following instructions provided by the manufacturer. The extension products from the sequencing reactions were purified by gel filtration with Sephadex G-50 (GE Healthcare, Pittsburgh, PA) using spin columns in a FiltrEX 96 well filter plate (Corning, Corning, NY) to remove unincorporated dye terminators. The final DNA sequence reading was performed on an Applied Biosystems 3730xl DNA Analyzer by the Genomics Facility in the Biotechnology Resource Center, Cornell University, Ithaca, NY. The COI fragments from the thrips individuals were sequenced in both strands.

DNA sequences from thrips individuals were assembled and aligned to identify single nucleotide polymorphisms (SNPs), using the DNASTAR Lasergene software suite (DNASTAR, Madison, WI). For analysis of the phylogenetic relationships of the *T. tabaci* COI haplotypes identified in this study and from public databases, a BLASTN search [21] against the NCBI GenBank [22] nucleotide database was performed to obtain COI sequences that had been deposited in the GenBank. For the identical sequences from different GenBank accessions, only one accession was selected and used for phylogenetic sequence analysis. Phylogenetic analyses were performed using the maximum likelihood method based on the Jukes-Cantor model [23] and molecular evolutionary genetics analysis software MEGA 5.2 [24]. For analysis of host plant association and seasonal changes in thrips population genetics, haplotype networks for the thrips populations were constructed based on statistical parsimony [25], using the software TCS 1.21 [26]. For analysis of *T. tabaci* populations from cabbage and onion fields collected during the two seasons, the population fixation index *F*_ST_ was calculated using the software ARLEQUIN 3.5.1.2 [27] for all pairwise population comparisons at *P*<0.01.

## Results

### Thrips tabaci *COI* haplotypes

From 565 *T. tabaci* individuals sequenced, 10 SNP sites were identified within the 655 bp COI fragment (excluding the primer sequence regions) (Figure S1), yielding eight haplotypes, NY-HT1 to NY-HT8 (GenBank accession numbers: KF036290 to KF036297, respectively). Five haplotypes (NY-HT1, NY-HT2, NY-HT3, NY-HT5 and NY-HT8) were found in samples collected from onion and seven haplotypes (NY-HT1, NY-HT2, NY-HT3, NY-HT4, NY-HT5, NY-HT6 and NY-HT7) were found in samples collected from cabbage (Tables S1 and S2). The SNPs in the 655 bp fragment were all synonymous substitutions, except an A/G substitution at nucleotide position 98 (Figure S1), resulting in a change between two amino acid residues, isoleucine and valine.

**Figure 2:**
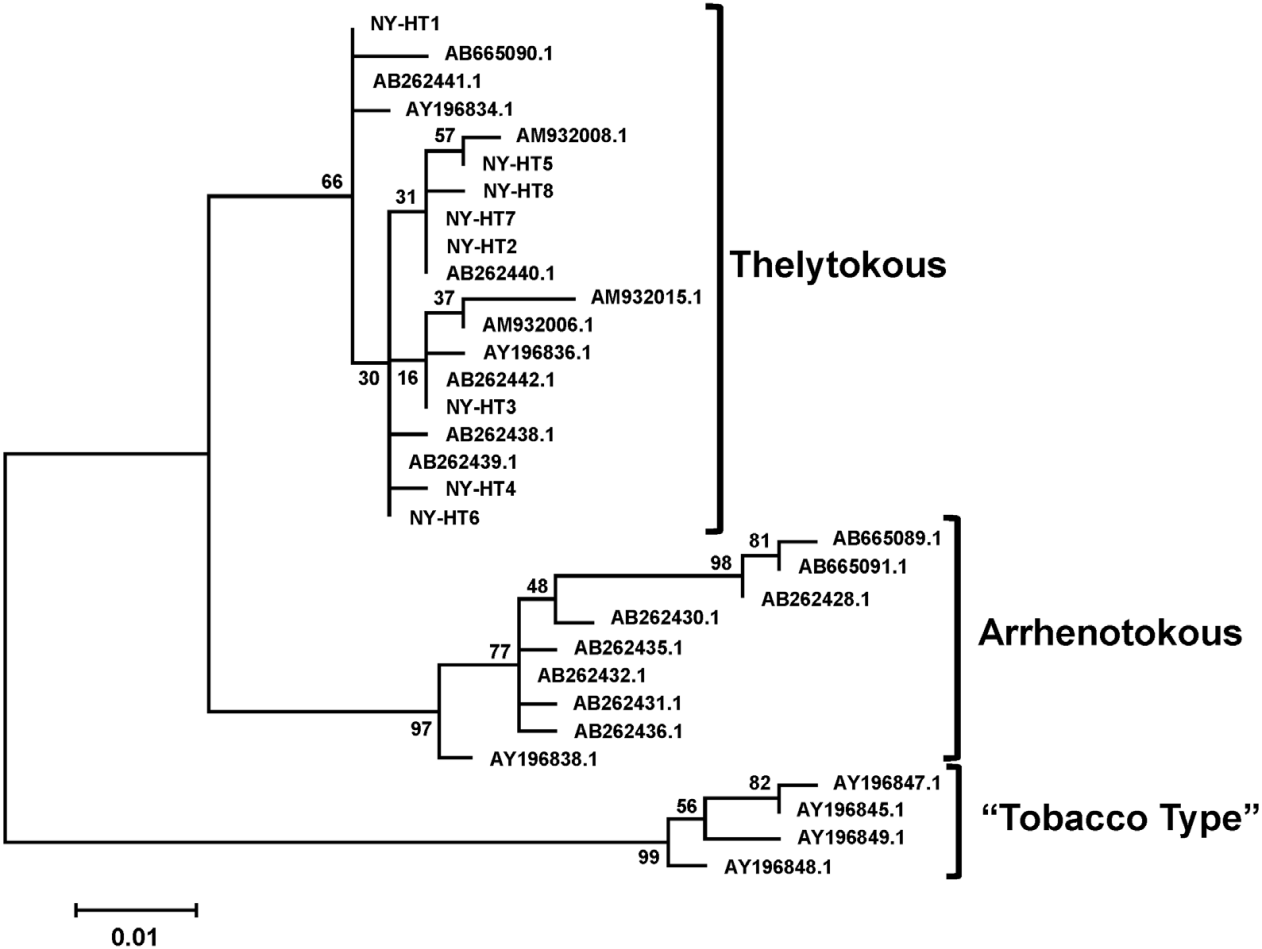
Phylogenetic tree of *Thrips tabaci* COI sequences collected from onion and cabbage fields in New York (NY-HT1 to NY-HT8) relative to others with known reproductive mode. The tree was generated by the maximum likelihood method based on the Jukes-Cantor model [23] using MEGA5 [24]. Sequence positions containing gaps and missing data were eliminated and bootstrap values (percentages of 1000 replicates) were shown next to the branches.

Nucleotide polymorphisms at positions 355 and 515 (Figure S1) differentiate the two reproductive types of *T. tabaci,* arrhenotoky and thelytoky [4]. In all eight haplotypes, the nucleotides at 355 and at 515 were G and T, respectively, indicating that the *T. tabaci* individuals sampled in this study were all thelytokous (Figure S1). Further analysis of the eight haplotypes with the COI sequences from known reproductive types of *T. tabaci* showed that the eight haplotypes were in the same cluster with the thelytokous type (Figure 2).

### Phylogenetic analysis of *Thrips tabaci* from different geographic regions and host plants

**Figure 3:**
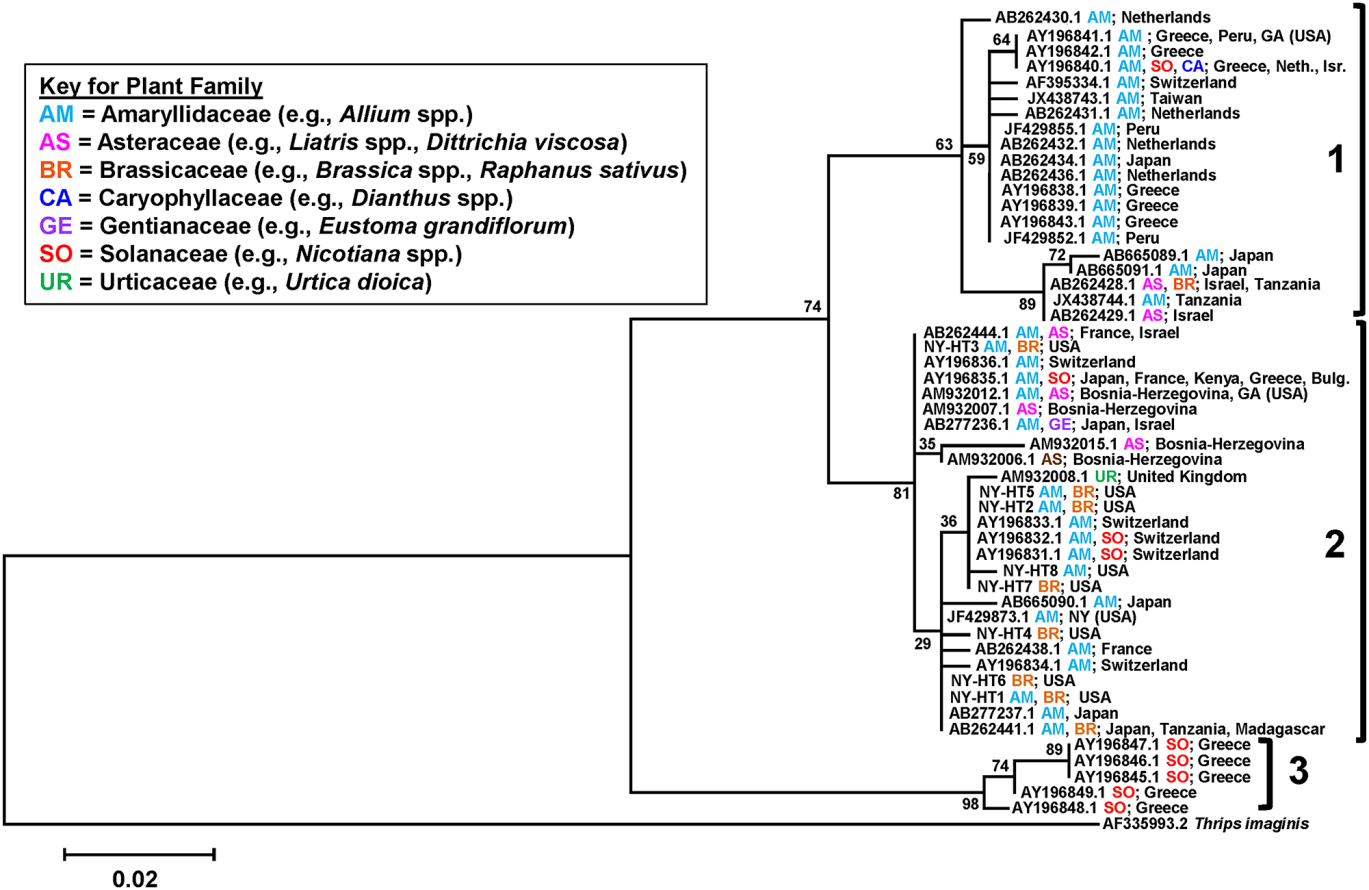
Phylogenetic tree of *Thrips tabaci* COI sequences collected from onion and cabbage fields in New York (NY-HT1 to NY-HT8) relative to others collected from an array of host plants from around the world. The Maximum Likelihood method was used for constructing the phylogenetic tree. Genbank accession number, host plant and country where the specimen originated are included for each entry in the tree. *Thrips imaginis* was included as an outgroup and the “tobacco type” as well. Bootstrap values from 1000 replicates are shown above branches.

From GenBank, in total 133 COI sequences were found under *T. tabaci* by blast search as of Feb. 13, 2013. In these sequence accessions, 82 accessions were associated with geographic region and plant-host information of *T. tabaci* origins. Among the 82 sequences, 43 sequences remained after redundant (identical) sequences were combined and were chosen for this study. A phylogenetic tree of the eight *T. tabaci* COI haplotypes and the 43 sequences from GenBank was generated with the COI sequence from *T. imaginis* as an outgroup, by maximum likelihood analysis (Figure 3). The phylogenetic tree showed three distinct clades for the *T. tabaci* haplotypes. Haplotypes in Clade 1 and Clade 2 had a high degree of host plant diversity and geographical representation - seven plant families over five continents. Clade 1 includes haplotypes from the arrhenotokous strain and Clade 2 includes haplotypes from the thelytokous strain. Interestingly, Clade 1 shows a subdivision with bootstrapping support at 63%. The haplotypes in one branch are associated with three host plant families, but the haplotypes from the other branch were primarily found in host plants in the family Amaryllidaceae, except one haplotype found from two additional host plant families (Figure 3). Clade 3 was composed of haplotypes that have so far only been found in individuals from tobacco in Greece and is known as the “tobacco strain” [7].

### Population structure of *Thrips tabaci* in onion fields and cabbage fields

**Figure 4:**
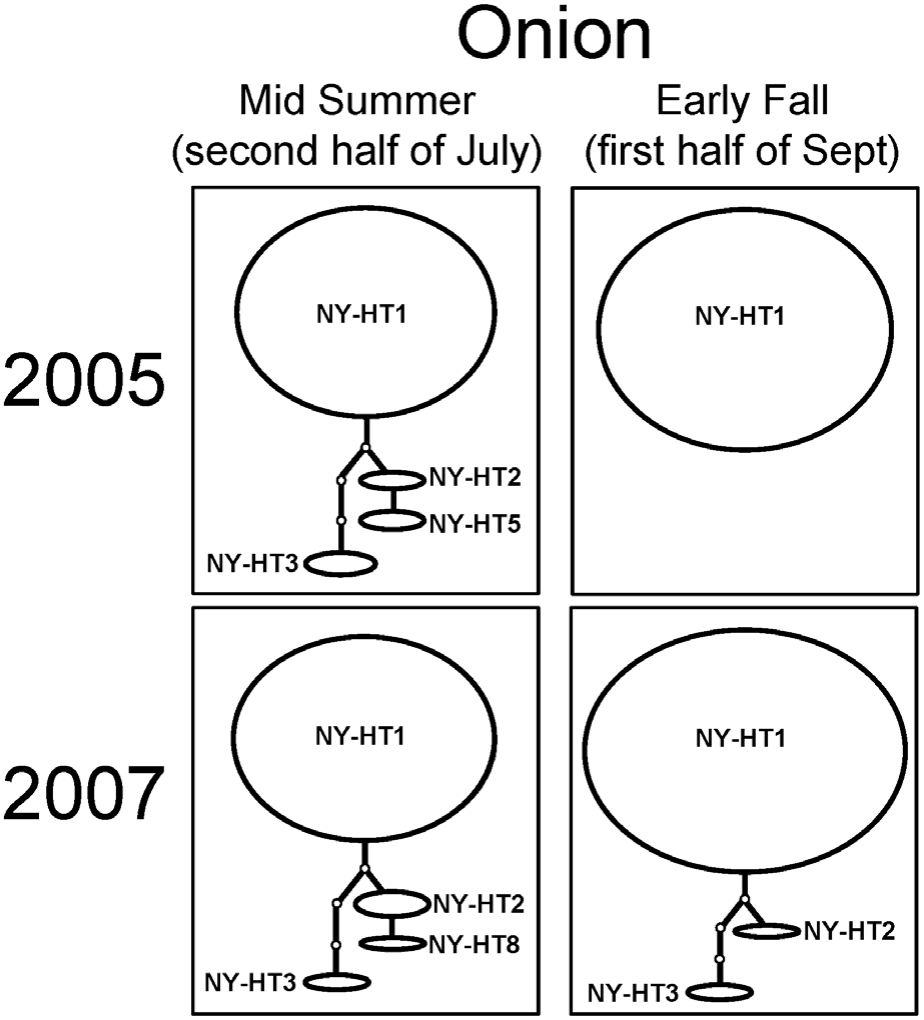
Haplotype networks for *Thrips tabaci* COI sequences from populations collected from onion, *Allium cepa*, during mid-summer and early fall in New York in 2005 and 2007. Each ellipse represents a certain haplotype (NY-HT1 to NY-HT8) and its size is proportional to the number of individuals from the population that it represents. Branches connecting the haplotypes represent the number of inferred mutations.

**Figure 5:**
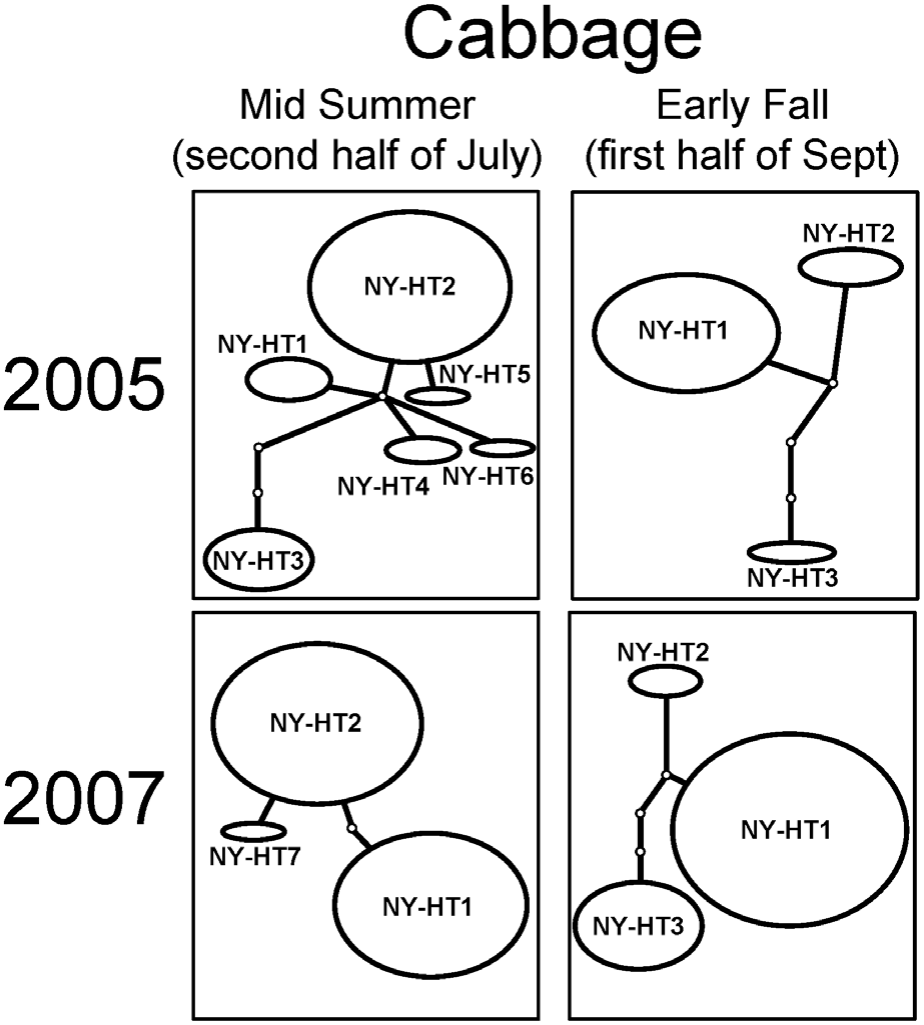
Haplotype networks for *Thrips tabaci* COI sequences from populations collected from cabbage, *Brassica oleracea*, during mid-summer and early fall in New York in 2005 and 2007. Each ellipse represents a certain haplotype (NY-HT1 to NY-HT8) and its size is proportional to the number of individuals from the population that it represents. Branches connecting the haplotypes represent the number of inferred mutations.

Although eight haplotypes were identified in *T. tabaci* populations in onion and cabbage fields in New York, the haplotypes NY-HT1, NY-HT2 and NY-HT3 accounted for the vast majority of the individuals sampled, with NY-HT1 being the most abundant. *Thrips tabaci* COI haplotype networks indicated that the haplotype composition in mid-summer and early fall remained the same in years 2005 and 2007 (Figure 4). The majority of individuals collected from onion were NY-HT1, accounting for 92% and 88% of the total in mid-summer in 2005 and 2007, respectively, and 100% and 96% of the total in early fall in 2005 and 2007, respectively (Figure 4; Table S1). In contrast, haplotype networks of *T. tabaci* populations collected from cabbage fields showed a population structure changing dynamically from mid-summer to early fall (Figure 5). In *T. tabaci* populations from cabbage in mid-summer, the haplotype NY-HT2 was abundant, accounting for 58% and 52% of the total in 2005 and 2007, respectively, but in early fall this haplotype decreased to 15% and 7% of the total in 2005 and 2007, respectively (Figure 5; Table S2). A lower percentage of the individuals collected from cabbage in mid-summer were haplotype NY-HT1 (12% and 46% in 2005 and 2007, respectively), but a majority in early fall were NY-HT1 (81% and 66% in 2005 and 2007, respectively) (Figure 5; Table S2).

The population structural change of *T. tabaci* from mid-summer to early fall in cabbage fields also was clearly indicated by the *F*_ST_ values (Table 1). The pairwise *F*_ST_ values between the mid-summer and early-fall populations in cabbage collected in 2005 and 2007 were 0.307 (*P*<0.01) and 0.244 (*P*<0.01), respectively. However, the *F*_ST_ values for the populations in onion were very low, 0.033 and 0.034 in 2005 and 2007, respectively, showing minimal population structural change between the two seasons. Pairwise comparisons of *F*_ST_ values between the *T. tabaci* populations indicated that *T. tabaci* populations in cabbage and onion had different genetic structures (Table 1). The greatest difference was between the mid-summer populations in cabbage and the early-fall populations in onion with *F*_ST_ values between 0.452 (*P*<0.01) and 0.551 (*P*<0.01).

**Table 1:** Pairwise *F*_ST_ values^1^ for *Thrips tabaci* populations collected from cabbage and onion fields at two times during the season in New York (USA).

Year	*T. tabaci* population	Cabbage/Mid-summer	Cabbage/Early fall	Onion/Mid-summer
2005	Cabbage/Early fall	0.307*		
	Onion/Mid-summer	0.472*	0.040	
	Onion/Early fall	0.551*	0.198*	0.033
2007	Cabbage/Early fall	0.244*		
	Onion/Mid-summer	0.282*	0.148*	
	Onion/Early fall	0.452*	0.209*	0.034

1 *F*_ST_ values were calculated using ARLEQUIN 3.5.1.2.

*P value<0.01, calculated by permutation test.

## Discussion

Since Brunner et al.’s [7] phylogenic analysis that identified three major genetic lineages for *T. tabaci* (i.e., Leek 1, Leek 2 and Tobacco strains), numerous similar analyses from around the world have followed [4], [6], [16], [17], [28]. All *T. tabaci* in our study were from a single leek clade that included individuals collected from an array of hosts representing seven plant families and multiple locations spanning five continents (clade number 2 in Figure 3). Like many other phylogenetic studies, *T. tabaci* from New York in our study differed substantially from those identified as the “tobacco strain” (clade number 3 in Figure 3). All haplotypes in our study were from the same lineage as those that reproduce via thelytoky. *Thrips tabaci* that reproduce via thelytoky differ genetically from those that reproduce via arrhenotoky [4], [16]. In regions of New York, North Carolina (USA) and Japan, both thelytokous and arrhenotokous populations of *T. tabaci* collected from onion exist [3]–[5]. In North Carolina, arrhenotokous populations were found only in the mountains in the western region, while thelytokous ones were found at lower elevations in the central region of the state. In New York, arrhenotokous and thelytokous populations were identified in nearby onion fields within each of the major onion-producing regions. In Japan, thelytokous and arrhenotokous populations were documented from the same field. Subsequent studies on *T. tabaci* populations in Japan confirmed that populations of sexually and asexually reproducing populations of *T. tabaci* coexist within the same crop field [29].

*Thrips tabaci* populations changed considerably during the season in cabbage fields, but not in onion fields (Table 1; Figures 4 and 5). *Thrips tabaci* populations in onion fields were dominated by a single haplotype (NY-HT1) during mid-summer and early fall, whereas populations in cabbage fields included several common haplotypes that changed considerably from mid-summer to early fall. The haplotype NY-HT2 was noticeably abundant in cabbage fields in mid-summer, but became a minor type in early fall (Figure 5). Despite the proximity of onion and cabbage fields in the western New York landscape, *T. tabaci* populations appeared to differ seasonally within each cropping system. The *T. tabaci* population in cabbage fields underwent a genetic structural change from mid-summer to early fall, suggesting that some individuals such as those with COI haplotype NY-HT1 outcompete others and ultimately establish more successfully later in the season on cabbage plants or that dispersal and colonization patterns differ within cabbage and onion cropping systems, or perhaps both of these phenomena occur.

While onion and cabbage fields were grown in proximity (Figure 1), habitats immediately adjacent to these crops differed. In New York, onion is grown in muck cropping systems, which are essentially islands in the landscape bordered by woods or weedy areas known to harbor *T. tabaci* [30], [31]. Onion fields are rarely bordered by other cultivated hosts for *T. tabaci* such as alfalfa and small grains, both of which are grown adjacent to cabbage and are speculated to contribute to early-season infestations in cabbage fields in New York [32]. Although dispersal behavior of *T. tabaci* over long distances is poorly understood, if dispersal occurs over long distances (i.e., several km or more), it is possible that *T. tabaci* populations from onion may colonize cabbage and other crops late in the season following onion harvest in August and September. If so, this may explain the late-season occurrences of NY-HT1 in cabbage fields.

Although *T. tabaci* has a wide host range [33], host-associations involving onion/leek and tobacco have been documented for *T. tabaci* [7]. Our results provide additional evidence for the occurrence of host associations between different genetic lineages of *T. tabaci* and other cultivated hosts. The extent to which *T. tabaci* populations restrict themselves to specific crop hosts in space and time will likely influence the population dynamics related to the development and spread of traits such as insecticide resistance within and among different cropping systems in the same area. Future studies investigating population structures of *T. tabaci* on their major hosts in these ecosystems through time are needed to better understand host-plant utilization and its potential implications for population management.

## Supporting Information

Figure S1 **DNA sequence of the 655 bp** ***COI*** **fragment from** ***Thrips tabaci*** **collected in New York.** Eight COI haplotypes were identified from 565 *T. tabaci* individuals, based on the SNP site (R = A or G; Y = C or T). The nucleotides G and T boxed are specific for thelytokous *T. tabaci* [4].
(TIF)

Table S1 **Frequency of** ***Thrips tabaci*** **haplotypes collected from onion fields,** ***Allium cepa*** **L, during mid-summer and early fall in western New York in 2005 and 2007.**
(DOCX)

Table S2 **Frequency of** ***Thrips tabaci*** **haplotypes collected from cabbage fields,** ***Brassica oleracea*** **, during mid-summer and early fall in western New York in 2005 and 2007.**
(DOCX)
