# Supplementary figures and images for: Seasonal Changes in Thrips tabaci Population Structure in Two Cultivated Hosts
Source: PLoS One. 2014 Jul 3;9(7):e101791. doi: 10.1371/journal.pone.0101791 (PMC4081722; doi:10.1371/journal.pone.0101791)

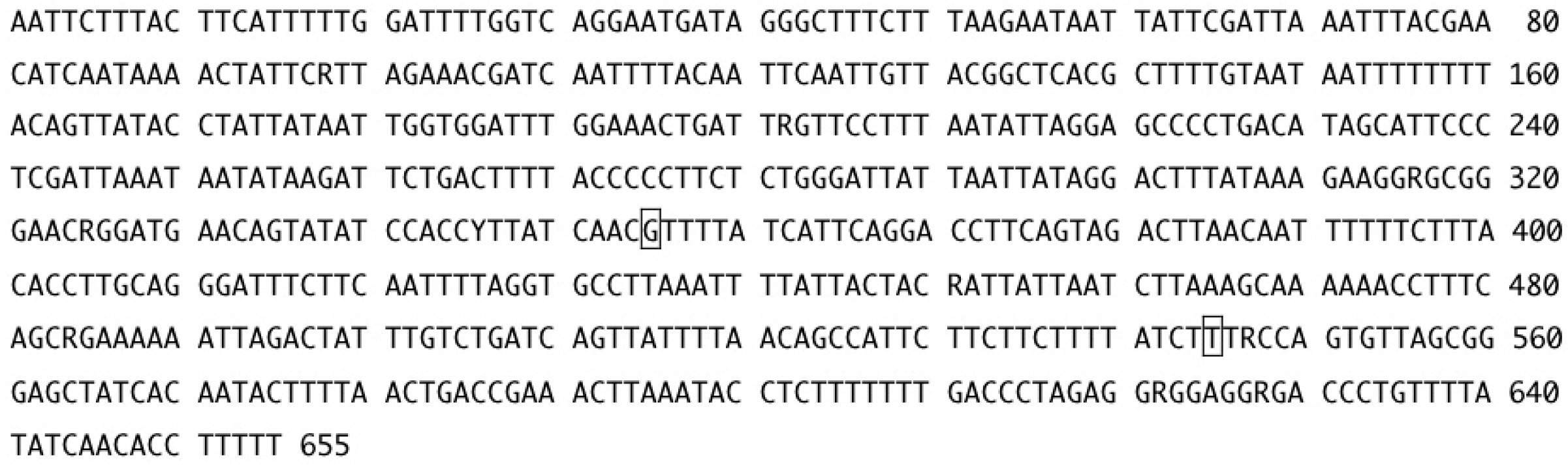

Supplement: Figure S1 — DNA sequence of the 655 bp COI fragment from Thrips tabaci collected in New York. Eight COI haplotypes were identified from 565 T. tabaci individuals, based on the SNP site (R = A or G; Y = C or T). The nucleotides G and T boxed are specific for thelytokous T. tabaci [4]. (TIF) [file pone.0101791.s001.tif]
