# Supplementary material for: Seasonal Changes in Thrips tabaci Population Structure in Two Cultivated Hosts
Source: PLoS One. 2014 Jul 3;9(7):e101791. doi: 10.1371/journal.pone.0101791 (PMC4081722; doi:10.1371/journal.pone.0101791)
Supplement: Table S1 — Frequency of Thrips tabaci haplotypes collected from onion fields, Allium cepa L, during mid-summer and early fall in western New York in 2005 and 2007. (DOCX) [file pone.0101791.s002.docx]

Table S1. Frequency of *Thrips tabaci* haplotypes collected from onion fields, *Allium cepa* L, during mid-summer and early fall in western New York in 2005 and 2007.

|  | |  | | | | | Frequency of haplotype | | | | | | | | |
| --- | --- | --- | --- | --- | --- | --- | --- | --- | --- | --- | --- | --- | --- | --- | --- |
| Year | Season | | Date Collected | County (Field) | GPS Coordinates |  | HT1 | HT2 | HT3 | HT4 | HT5 | HT6 | HT7 | HT8 |  |
| 2005 | Mid-summer | | 15 July | Wayne (1) | 43.173268,-77.078930 |  | 23 | 1 | 1 | 0 | 1 | 0 | 0 | 0 |  |
|  |  | | 15 July | Yates (1) | 42.712856,-77.194104 |  | 18 | 0 | 2 | 0 | 0 | 0 | 0 | 0 |  |
|  |  | | 29 July | Orleans (1) | 43.140499,-78.127534 |  | 20 | 0 | 0 | 0 | 0 | 0 | 0 | 0 |  |
|  |  | |  |  |  |  |  |  |  |  |  |  |  |  |  |
|  | Early fall | | 8 Sept | Wayne (1) | 43.173268,-77.078930 |  | 24 | 0 | 0 | 0 | 0 | 0 | 0 | 0 |  |
|  |  | | 18 Sept | Yates (1) | 42.712856,-77.194104 |  | 16 | 0 | 0 | 0 | 0 | 0 | 0 | 0 |  |
|  |  | | 19 Sept | Orleans (2) | 43.140765,-78.125753 |  | 23 | 0 | 0 | 0 | 0 | 0 | 0 | 0 |  |
|  |  | |  |  |  |  |  |  |  |  |  |  |  |  |  |
| 2007 | Mid-summer | | 18 July | Wayne (2) | 43.171257,-77.077793 |  | 30 | 0 | 0 | 0 | 0 | 0 | 0 | 0 |  |
|  |  | | 26 July | Yates (1) | 42.712856,-77.194104 |  | 17 | 6 | 1 | 0 | 0 | 0 | 0 | 1 |  |
|  |  | | 23 July | Orleans (3) | 43.132967,-78.117278 |  | 23 | 1 | 1 | 0 | 0 | 0 | 0 | 0 |  |
|  |  | |  |  |  |  |  |  |  |  |  |  |  |  |  |
|  | Early fall | | 19 Sept | Wayne (2) | 43.171257,-77.077793 |  | 29 | 0 | 1 | 0 | 0 | 0 | 0 | 0 |  |
|  |  | | 5 Sept | Yates (1) | 42.712856,-77.194104 |  | 26 | 1 | 0 | 0 | 0 | 0 | 0 | 0 |  |
|  |  | | 5 Sept | Orleans (4) | 43.138745,-78.126526 |  | 27 | 0 | 1 | 0 | 0 | 0 | 0 | 0 |  |
