# Supplementary material for: Seasonal Changes in Thrips tabaci Population Structure in Two Cultivated Hosts
Source: PLoS One. 2014 Jul 3;9(7):e101791. doi: 10.1371/journal.pone.0101791 (PMC4081722; doi:10.1371/journal.pone.0101791)
Supplement: Table S2 — Frequency of Thrips tabaci haplotypes collected from cabbage fields, Brassica oleracea , during mid-summer and early fall in western New York in 2005 and 2007. (DOCX) [file pone.0101791.s003.docx]

Table S2. Frequency of *Thrips tabaci* haplotypes collected from cabbage fields, *Brassica oleracea*, during mid-summer and early fall in western New York in 2005 and 2007.

|  | |  | | | | | Frequency of haplotype | | | | | | | | |
| --- | --- | --- | --- | --- | --- | --- | --- | --- | --- | --- | --- | --- | --- | --- | --- |
| Year | Season | | Date Collected | County (Field) | GPS Coordinates |  | HT1 | HT2 | HT3 | HT4 | HT5 | HT6 | HT7 | HT8 |  |
| 2005 | Mid-summer | | 28 July | Ontario (1) | 42.76847,-77.009053 |  | 0 | 24 | 0 | 1 | 0 | 0 | 0 | 0 |  |
|  |  | | 29 July | Ontario (2) | 42.917291,-77.064092 |  | 0 | 17 | 1 | 4 | 1 | 1 | 0 | 0 |  |
|  |  | | 21 July | Orleans (1) | 43.109803,-78.117857 |  | 9 | 2 | 14 | 0 | 0 | 0 | 0 | 0 |  |
|  |  | |  |  |  |  |  |  |  |  |  |  |  |  |  |
|  | Early fall | | 8 Sept | Ontario (2) | 42.917291,-77.064092 |  | 1 | 4 | 0 | 0 | 0 | 0 | 0 | 0 |  |
|  |  | | 8 Sept | Yates (1) | 42.756167,-77.158077 |  | 20 | 0 | 1 | 0 | 0 | 0 | 0 | 0 |  |
|  |  | |  |  |  |  |  |  |  |  |  |  |  |  |  |
| 2007 | Mid-summer | | 25 July | Ontario (3) | 42.885524,-77.027518 |  | 13 | 17 | 0 | 0 | 0 | 0 | 0 | 0 |  |
|  |  | | 26 July | Yates (2) | 42.781157,-77.071538 |  | 25 | 3 | 0 | 0 | 0 | 0 | 0 | 0 |  |
|  |  | | 17 July | Genesee (1) | 43.058604,-78.141353 |  | 1 | 24 | 0 | 0 | 0 | 0 | 1 | 0 |  |
|  |  | |  |  |  |  |  |  |  |  |  |  |  |  |  |
|  | Early fall | | 13 Sept | Ontario (4) | 42.88903,-77.080743 |  | 13 | 0 | 16 | 0 | 0 | 0 | 0 | 0 |  |
|  |  | | 13 Sept | Yates (3) | 42.766348,-77.067043 |  | 24 | 4 | 1 | 0 | 0 | 0 | 0 | 0 |  |
|  |  | | 5 Sept | Genesee (2) | 43.061818,-78.153884 |  | 20 | 2 | 7 | 0 | 0 | 0 | 0 | 0 |  |
